# Linkages between social and financial performance: Evidence from Sub-Saharan Africa microfinance institutions

**DOI:** 10.1371/journal.pone.0261326

**Published:** 2022-03-01

**Authors:** Amidou Ayinla Akangbe Fadikpe, Richard Danquah, Mohammed Aidoo, Dejene Adugna Chomen, Richard Yankey, Xie Dongmei

**Affiliations:** 1 College of Economics, Fujian Agriculture & Forestry University, Fuzhou, Fujian, China; 2 School of Insurance and Economics, University of International Business and Economics, Beijing, China; 3 School of Economics, Henan University, Kaifeng, China; 4 School of International Trade and Economics, University of International Business and Economics, Beijing, China; 5 College of Life Science, Fujian Agriculture & Forestry University, Fuzhou, Fujian, China; University of Almeria, SPAIN

## Abstract

Microfinance Institutions provide financial services to low-income clients and the poor who are excluded from formal financial institutions. Hence, the sustainability of microfinance institutions (MFIs) remains essential. This study examines the relationship between social and financial performance and whether there is a trade-off between both objectives after the 2008 global financial crisis. The study used 735 observations from 105 Microfinance Institutions across 26 countries in Sub-Saharan Africa from 2011 to 2017 and employed the Generalized Method of Moment and Seeming Unrelated Regression for the analyses. The results indicate that increasing the number of customers [breadth of outreach increased the financial performance (return on equity)]. The result also showed that the Percentage of Female Borrowers contributes to the sustainability of Microfinance Institutions due to their higher loan repayment rate than males. In addition, our results document a trade-off between the Depth of Outreach and Operational Self-Sustainability among Microfinance Institutions. The study recommends the following: 1) Microfinance institutions should purposefully increase credit facilities extended to female borrowers since that will make them sustainable. 2) Governments in Sub-Saharan African countries should provide increased financial support in the form of subsidies and tax holidays to Microfinance Institutions operating in very deprived areas, and 3) Management of Microfinance institutions on the continent should regularly re-train and upgrade their staff capacity to effectively assess and manage customers before and after extending credit to them to sustain the industry.

## Introduction

Microfinance has been considered a developmental tool to reduce poverty by providing long-term assistance to the poor in many developing countries, particularly Sub-Saharan Africa (SSA). It offers financial services to clients/customers who lack access to mainstream banks/other formal financial service providers, based on its structural structure, mission, and approach/methodology [1]. Microfinance institutions (MFIs) in SSA, like those in other regions, provide low-income people with loans, money transfers, savings, insurance, and other financial services. In SSA, microfinance institutions are expanding their operations faster, thus positioning them to be among the most productive in the world in terms of the number of savings and borrowers. Furthermore, microfinance institutions are currently being used in SSA as developmental tools to help people get out of poverty [2].

MFIs have traditionally received funding from non-profits (social performance) organizations such as international donors, grants, donations, government, and subsidies to support their various missions of poverty alleviation. However, donations and subsidies alone cannot support the expansion and growth of the MFIs industry [3], and some policymakers and practitioners emphasize the long-term viability of donor funds support. This situation has led to the appearance of profit-oriented (financial performance) organizations such as commercial MFIs whose goal is to be financially sustainable by making profits. Achieving this dual mission (social performance and financial performance) presents a challenge for MFIs. They often focus on their financial self-sufficiency or profitability, which takes them away from their social mission. Conversely, MFIs that focus on the sole objective of achieving their social mission can be threatened in their sustainability and profitability.

There are four main areas to measure MFIs performance: sustainability, outreach, portfolio quality, and efficiency [4] and, these areas are categorized into social performance and financial performance. Outreach and percentage of female borrowers (PFB) capture social performance while sustainability, portfolio quality, and efficiency capture financial performance. This study considers two important aspects of outreach as a measure of social performance. First, depth of outreach implies increased service to the poor [5]. Second, breadth of outreach refers to the number of clients served by MFIs. A larger number of borrowers imply a greater breadth of outreach. This distinction shows the importance of different effects of outreach on different types of financial performance in literature. Among the indicators to measure financial performance considered in the study are Operational Self-Sufficiency (OSS)—to provide the services profitably to ensure MFIs are sustainable; and profitability–measured as Return on Asset (ROA) and Return on Equity (ROE). Aid alone, such as grants, subsidies, and other benefits to MFIs, cannot help them be sustainable; therefore, according to some decision-makers, the pursuit of profitability is very important to MFIs. This situation has led to some tensions in the MFIs industry because focusing on financial performance may impact their social mission [6].

The study tries to narrow the understanding of the trade-off of MFIs performance in the literature regarding social performance and financial performance after the global financial crises. This is because most of these MFIs rely on NGOs and governments to support their operations. Additionally, the study used Percentage of Female Borrowing (PFB) as a measure of social performance since women are generally poorer than men [7], and more vulnerable. The linkage between social and financial performance is more complicated than we may think, and there is evidence that it is not necessarily positive or negative. For example, Rahman [8] found a positive relationship between social and financial performance in Bangladesh. Kamaluddin and Kasim [9], on the other hand, discovered no link between social and financial performance in Malaysia. Similarly, although a few studies have examined the relationship between social and financial performance in SSA, the results are inconclusive [4, 5].

Furthermore, earlier research has utilized fixed and random effect estimators to measure the correlation between MFI social performance and financial performance. Still, these estimators are unable to solve the problem of endogeneity bias in panel data. This demonstrates that a methodological gap exists in the literature. However, this study applies a panel generalized method of moments (GMM) estimators that are versatile in dealing with endogeneity biases in socio-economic data [10]. Therefore, to address these gaps in the literature, the main objective of this study is to examine the relationship between social performance and the financial performance of MFIs in SSA countries.

The rest of the study is organized as follows. The second section provides the literature review, and the third section describes the data, variables, and methodologies used in our research. The fourth section presents and discusses the results of the empirical findings. The fifth section concludes our study.

## Literature review

### Theoretical literature review

Several scholars have attempted to justify the link between MFIs’ social and financial performance in various ways. Conning [11] was among the first to focus on the trade-off between social and financial performance in MFIs. He emphasized the importance of MFIs to increase loan access to individuals who do not have adequate collateral. However, with less collateral, monitoring is even more critical, and to minimize moral hazard, the borrower-lender relationship could be monitored. Other researchers connect social and financial performance in various ways. According to Copestake [12], MFIs can simultaneously attain social and financial performance. Copestake [12] posits that lowering costs can result in higher returns on assets, thus allowing MFIs to hire more competent officers to drive their social objectives.

Moreover, Waddock and Graves [13] argue that financial stability of MFIs allow access to “resources and capabilities,” which can then be utilized to fulfill social objectives. When MFIs are financially unstable, on the other hand, financial stability will take priority over social performance. Similarly, Copestake [12] discovered a trade-off between MFIs’ social and financial performance. One explanation for this is that increasing interest rates, for example, can improve short-term financial performance while also putting additional stress on clients, leading to social problems.

Likewise, Armendáriz and Murdoch [14] argue that both current social and financial performances influence the future social performance of MFIs. Furthermore, the successful fulfillment of social performance increases the demand for micro-loans. On the other hand, excellent financial performance is necessary for future sustainability and expansion, which increases the number of prospective customers served and thus has been a primary priority for socially focused MFIs. Various theories or approaches in microfinance explore the relationship between social and financial performance, such as the institutionalist approach, the welfarist approach, and the trade-off approach.

#### Institutionalist approach

Institutionalists argue that microfinance has to improve its financial performance to effectively reduce poverty [15]. They believe that MFIs have to focus more on their sustainability. Institutionalists MFIs are considered to be not-for-profit organizations that are gradually becoming regulated commercial institutions [16]. Defenders of the approach (institutionalists) show the importance of MFIs viability by reducing operational costs and covering costs from revenues. In addition, others argue that commercialized MFIs develop better even without subsidies to meet the growing demand of their clients. MFIs that achieve financial sustainability better serve poor entrepreneurs without the constraints imposed by donor budgets [17].

#### Welfarist approach

According to the welfarist, MFIs need subsidies to support high operational costs to focus on the poor and reduce poverty. Welfarists are concerned about the social mission of MFIs, and social investors are willing to sacrifice financial returns to invest in MFIs with a social mission. They argue that institutions that focus on serving the poorest should not be concerned about their financial viability. The welfarist approach is based on the assumption that MFIs can achieve sustainability without self-sufficiency [18]. In other words, commercial (institutionalists) microfinance services have a limited contribution to poverty reduction [19].

#### Trade-off theory

MFIs striving to achieve the dual goal of social and financial performances are faced with a trade-off between debt and equity in their capital structure. The optimal combination of debt and equity in MFIs largely determines whether they will be focused on achieving social performance to the neglect of financial performance or vice versa or focusing on achieving both social and financial performance. Trade-off theorists suggest that optimal capital structure can be determined by creating equilibrium between corporate tax benefits and cost associated with debts, thus assuming costs and benefits associated with using debts against equities. The main objective of this study is to examine the relationship between social performance and the financial performance of MFIs in SSA countries. However, this relationship between social performance and financial performance of MFIs hinges on their capital structure, indicating the type of objective or performance (social and or financial) they would want to achieve.

Social performance focuses on improving the living standards of the poor. The importance of MFIs is often demonstrated by their ability to respond to the needs of the poor in the short term and their impact on the poor population. However, the financial performance focuses primarily on the profitability and self-sufficiency of MFIs. A significant change that the MFIs sector has faced in the last decade is determining whether there is a relationship between social and financial performances. Some results in literature reveal the existence of trade-off in achieving the dual objective, however, the results are mixed in positive, negative, and neutral depending on the study [20]. There may be a trade-off between outreach (social performance) and sustainability (financial performance). Evidence suggests that increasing outreach by lending to the poor reduces the sustainability and efficiency of MFIs. Additionally, funding sources and lending strategies can have significant roles in MFIs’ decision to establish an optimal capital structure, thereby influencing their financial and social performances. However, few empirical studies explain the determinants and nature of these trade-offs [21].

### Empirical literature review

With the continued development of microfinance, whether an MFI can maintain its basic social objectives while achieving financial viability remains a critical problem. The concept of a trade-off between financial and social performance has been studied in the literature, and empirical evidence on whether the outreach approach complements institutional sustainability has yielded conflicting outcomes. Furthermore, the results of the empirical studies extensively vary on the type of relationship between the financial and social objectives of MFIs. For instance, Quayes [5] studied the relationship between depth of outreach and financial sustainability. The result revealed a positive complementary relationship between financial sustainability and outreach depth. Similarly, in their research paper, Mosley and Hulme [22] found a positive relationship between social and financial performances.

Moreover, Fernando [23], while evaluating the effects of 39 MFIs (NGOs) in the world, demonstrates that the financial condition of the reformed MFIs improves without compromising their humanitarian objective. Similarly, according to a current study done in India by Navin and Sinha [24], the result indicated that lower loan sizes, as a proxy for outreach depth, make it easier for MFIs to attain financial sustainability. This suggests that MFIs can be financially sustainable while serving low-income clientele. Furthermore, Adhikary and Papachristou [25] empirically examined the trade-off between financial performance and outreach in a panel of 133 South Asian MFIs from 2003 to 2009, using random-effects estimation. The study found that the depth of outreach is positively related to financial performance, implying that financially sustainable expansion of microfinance can achieve social objectives at an acceptable level of credit risk.

Accordingly, Dewez and Neisa [26] carried out a study on synergies and trade-offs between social and financial performance based on 64 MFIs and 43 social performance indicators that take into account outreach, social responsibility, and client service, as well as a financial performance index that included 48 financial indicators. A significant positive association between social and financial performance was discovered using simple regression analysis. In addition, Cull et al. [16] showed strong evidence of a trade-off between sustainability and outreach, which focused on the role of institutional models in identifying the presence and size of these trade-offs. Also, Kar [27] studied the impact of profitability on the depth of outreach and found a significant positive relationship between MFIs size and average loan size, suggesting mission drift. Specifically, they discovered that operational self-sufficiency (OSS) and return on asset (ROA) were positively correlated with the gross loan-to-asset ratio, but not significantly so with OSS. Moreover, Berguiga et al. [28] used a panel from 2004 to 2015 to examine 67 MFIs in MENA nations, including 18 Islamic MFIs. They found that there is a trade-off between financial and social performance, despite whether the conventional MFI or Islamic.

On the other hand, several other microfinance research works have discovered a negative link between social and financial performance objectives. For example, Kipesha & Zhang [29] examined the presence of trade-offs between viability, profitability, and reach using a panel of 47 MFIs for four years from 2008 to 2011 using mixed market data and an unbalanced panel regression analysis model. They found that the focus on profitability harmed outreach to the poor. In the context of cultural considerations, Zainuddin et al. [30] studied the relationship between an MFI’s social and financial objectives. They discovered a negative correlation between outreach depth and long-term viability. They say that cultural factors determine the size of the trade-off.

Additionally, Hermes et al. [31] reported a trade-off between sustainability and outreach, using cost-effectiveness as a sustainability measure. According to their finding, outreach was found to be negatively related to MFI efficiency. Moreover, using OLS regressions on a global sample of 49 Islamic microfinance institutions (IMFIs) and 333 conventional microfinance institutions (CMFIs) between 1996 and 2012, Fersi and Boujelbene [32] analyze the drivers of social performance. According to their findings, in CMFIs, the number of active borrowers (NAB) harms social performance, as assessed by the average loan balance per borrower.

Apart from these two views, other empirical studies also suggest no relationship between social and financial performance. Ben Salem and Ben Abdelkader [33] examined the performance of 51 CMFIs and 14 IMFIs in the MENA countries during the period 2005–2010 using a non-parametric method (DEA). They found no considerable difference between IMFIs and CMFIs in terms of financial and social performance. Similarly, between 2008 and 2010, Bassem [34] examined the relationship between depth of outreach and financial performance on a sample of 64 MFIs in the MENA region and found no link between social and financial performance. Furthermore, Mohammad et al. [35] failed to demonstrate a relationship between social and financial performance in Bangladesh.

To sum up, although there are myriads of studies on social and financial performance relationships, empirical findings are unclear and inconsistent. Thus, the relationship between social and financial performance is still a hot topic of debate. Furthermore, most previous research focused on developing countries in general, and only a few focused on SSA. Moreover, majority of the existing studies on the linkages between social and financial performance used static panel models that cannot address the endogeneity issues. However, unlike most previous studies; this study uses the Generalized Method of Moment (GMM) to address the endogeneity problem. The current study also uses the Percentage of Female Borrowers as one of the proxies to measure the social performance of MFIs. Thus, by filling such gaps in the literature, this current study assesses the linkages between microfinance institutions social and financial performance by targeting some selected microfinance institutions in SSA after the 2008 global financial crisis.

## Data and methodology

### Data and sample description

The data for the study was obtained from the Microfinance Information Exchange (www.themix.org/mixmarket) and the World Bank database. The Microfinance Information Exchange (MIX) database primarily provides information on the operational, social, and financial performance of MFIs in all regions of the developing world. In selecting the sample, the goal was to have as many MFIs as possible with a rating of at least three diamonds after an audit of their financial statements. Data was collected for 105 MFIs over a seven-year duration from 26 countries from 2011 to 2017, culminating in 735 observations for the study. MFIs in this study comprise rural and community banks, cooperatives, credit unions, non-bank financial institutions (NBFI), and non-governmental organizations (NGOs).

### Variables definition

We used six variables to perform the confirmatory factor analysis. Three different variables, namely: Return on Asset (ROA), Return on Equity (ROE), and Operational Self-Sufficiency (OSS)], are used to measure financial performance. While ROE and ROA have been largely used as profitability measures in the literature, OSS is mostly used to measure sustainability in the microfinance industry. Three variables (depth of outreach, breadth of outreach, and Percentage of Female Borrower (PFB)) are used to measure the social performance of MFIs. The variables used are explained in Table 1 below.

**Table 1:** Variables definition. This table shows the variables used, their abbreviations, and how they are measured.

Variables	Abbreviations	Formula
**Performance**		
**Return on Assets**	ROA	(Net Operating Income, less Taxes)/Average Assets
**Return on Equity**	ROE	Net Operating Income, less Taxes) /Average Equity
**Operational Self Sufficiency**	OSS	Financial Revenue / (Financial expense on funding liabilities + Net impairment loss on gross loan portfolio + Operating expense)
**Breadth Outreach**	Breadth	Number of active women Borrowers / Adjusted Number of Active borrowers
**Depth Outreach**	Depth	Adjusted Average Loan Balance per Borrower / GNI per Capita
**Percentage of Female Borrowers**	PBF	Number of active female Borrowers/Number of active borrowers
**Capital Structure**		
**Equity / Capital**	Equity	Equity/ Total Assets
**Deposit**	Dep	Deposits / Total Assets
**Borrowings**	Borr	Non-deposits liabilities / Total Assets	
**Controls Variables**		
**Size**	Size	Natural Logarithm of Total Assets
**Asset Tangibility**	Tang	Asset Tangibility / Total Assets
**Regulation**	REG	Binary variable: 1 if the MFI is subject to prudential regulation, 0 otherwise
**Macro–Economic Variable**		
**Gross Domestic Product**	GDP	Annual growth rate of the GDP per capita of a country

Source: Authors’ compilation from the World Bank and the Mix market.

ROA is defined as the MFI’s net operating income divided by its assets. It is used as a profitability measure that measures an MFI’s ability to generate income from its assets [36]. It allows a comparison of MFIs performance and shows the investor expected return from an investment in the MFI. However, the return should cover the risk-free rate as well as a margin covering the MFIs systematic risk [37].

ROE is a percentage ratio that shows the amount of return earned by a microfinance institution’s equity [38]. This ratio is particularly important for commercialized MFIs because it shows the equity investor’s return on investment in the institution [36]. Also, it shows the efficiency of MFIs in generating profits from each unit of shareholder funds.

OSS refers to an MFI’s ability to cover all of its costs through its financial revenues [39]. This measure is an accurate way of measuring the financial viability of MFIs because it shows whether an MFI can cover its expenses through the balance of its operations. Sustainability is about providing microfinance services to clients in a profitable manner without depending on subsidies. Therefore, sustainable MFIs do not depend on subsidies to succeed or become profitable. Operational self-sufficiency, return on equity and return on assets have been used widely to measure the financial sustainability of MFIs [40].

In addition, the study employed depth of outreach which is measured by the average loan balance per borrower relative to GNI per capita [34–36]. It relates to the measurement of the poorest in society that MFIs have served; it shows that the poorer the customer, the higher the value per unit of profit. Many stakeholders are still interested in the depth of outreach measures, as financial inclusion, which extends financial services to the under-served poor, remains the most important element for their involvement in microfinance. This variable seems to be quite complicated [5]. However, this measure refers to the poverty level of MFIs clients [40, 41].

The breadth of outreach is measured by the number of borrowers, defined as the natural logarithm of the total number of active borrowers and the number of clients [42–44]. Breadth of outreach refers to the MFIs’ coverage and is generally measured by the number of clients served by the MFI. Breadth of outreach refers to the type or profile of clients served by the MFIs; it represents the scale of MFI operations [45, 46]. However, a high ratio of this variable indicates an increasing number of MFI operations and fewer poor clients being reached. This is consistent with institutionalists who believe that poverty reduction is related to the number of poor clients MFIs can serve [47].

Several gender studies have shown that women are generally poorer than men [7], making them more vulnerable. It has been demonstrated that women are significantly disproportionate among the extremely poor in many regions [3, 35, 39, 48]. Therefore, to reduce poverty, MFIs need to encourage women’s entrepreneurship and give them more space in their client portfolios. In addition, several studies have concluded that female borrowers repay better than male borrowers [49]. Women may be considered higher-risk borrowers because of their limited repayment capacity [50]; thus, lending to women is associated with lending to poorer borrowers. However, more female borrowers may imply better repayment rates, which would provide them with access to a wider range of MFI services. In addition, women in developing regions often face limited opportunities to access financial services, they will be more likely to have higher repayment rates in order to continue to be funded [51]. The percentage of female borrowers (PFB) as a proxy to encourage entrepreneurs as way to reduce the poverty level in Sub Saharan Africa countries.

Since our focus is on the reciprocal relationship between social performance and financial performance of MFIs, we constructed other control variables (equity, deposit, borrowings, firm size, asset tangibility, GDP, and Regulation) that could potentially explain the two performance measures in literature.

### Methodology

This study applied the two-step system Generalized Methods of Moment (System GMM) estimation with standard errors consistent with heteroskedasticity and autocorrelation. The system GMM, whose estimation is implemented by the xtabond2 command, assumes the non-correlation of first differences of instrument variables with fixed effects variables and thus builds upon the original and transformed equations [10].

Arellano and Bond [52], and Arellano and Bover [53] developed the Generalized Method of Moments, which can be employed for dynamic panel data with small time periods and many individual entities. According to Arellano and Bond [52], and [54], the system GMM introduces more instruments to improve the model’s efficiency. It makes instruments to be uncorrelated with fixed effects, as well as builds both original and transformed equations in the model, and it uses orthogonal deviations.

Following Blundell and Bond [55], the generalized method of moments model (GMM) of estimation is specified as follows:

$$Y_{it}={\emptyset Y}_{it-1}+{\beta X}_{it}^{\prime }+(\cap_{i}+\varepsilon_{it})$$ (1)

Where *Y*_*it*_ is the individual entity dependent variable at time t, *Y*_*it*−1_ is the entity lag dependent variable at time t, $X_{it}^{\prime }$ is the entity’s independent variables at time t, ∅ and β are the coefficients of the lag dependent and explanatory variables respectively. When the data has a short time “t”, and if Y is persistent, and Eq (1) is assumed to exhibit random walk, then the use of the difference GMM to estimate the model produces biased and inefficient estimates. In that regard, the system GMM is preferable. Blundell and Bond [54] posit that the system GMM is applicable in the above scenario because it involves using a greater number of moment conditions and expresses one equation in level form with the first difference as instruments and vice versa. The dynamic system GMM is thus specified as follows:

$$Y_{it}={\emptyset Y}_{it-1}+{\gamma Z}_{it}^{\prime }+{\beta X}_{it}^{\prime }+d_{t}+\varepsilon_{it}$$ (2)

Where, *X*′ are control variables, *d*_*t*_ is the time dummies and *ε*_*it*_ is the model error term. All other variables are as explained in Eq (1). By using Eq (2) for our estimation, our variables become as follows: Y_it_ is the dependent variables (ROA, ROE, and OSS) at time t; *Z*′ are explanatory variables (depth of outreach, breadth of outreach, and the percentage of female (borrowers (PFB)) at time t; *X*′ are control variables (firm size, asset tangibility, regulation, and GDP); *d*_*t*_ is the time dummies and *ε*_*it*_ is the model error term. Therefore, the three models estimated in this study are as follows:

$${ROA}_{it}=\alpha +{\emptyset ROA}_{it-1}+{\gamma_{1}Depth}_{it}^{\prime }+{\gamma_{2}Breadth}_{it}^{\prime }+{\gamma_{3}PFB}_{it}^{\prime }+{\beta_{1}Size}_{it}^{\prime }+{\beta_{2}Tang}_{it}^{\prime }+{\beta_{3}Reg}_{it}^{\prime }+{\beta_{4}GDP}_{it}^{\prime }+d_{t}+\varepsilon_{it}$$ (3)

$${ROE}_{it}=\alpha +{\emptyset ROE}_{it-1}+{\gamma_{1}Depth}_{it}^{\prime }+{\gamma_{2}Breadth}_{it}^{\prime }+{\gamma_{3}PFB}_{it}^{\prime }+{\beta_{1}Size}_{it}^{\prime }+{\beta_{2}Tang}_{it}^{\prime }+{\beta_{3}Reg}_{it}^{\prime }+{\beta_{4}GDP}_{it}^{\prime }+d_{t}+\varepsilon_{it}$$ (4)

$${OSS}_{it}=\alpha +\emptyset {OSS}_{it-1}+{\gamma_{1}Depth}_{it}^{\prime }+{\gamma_{2}Breadth}_{it}^{\prime }+{\gamma_{3}PFB}_{it}^{\prime }+{\beta_{1}Size}_{it}^{\prime }+{\beta_{2}Tang}_{it}^{\prime }+{\beta_{3}Reg}_{it}^{\prime }+{\beta_{4}GDP}_{it}^{\prime }+d_{t}+\varepsilon_{it}$$ (5)

Where ROA_it-1,_ ROE_it-1,_ OSS_it-1_ are lags of the dependent variables (Return on Asset, Return on Equity, and Operational Self Sustainability) respectively. Depth is Depth of Outreach, Breadth is Breadth of Outreach, PFB is the Percentage of Female Borrowers, Tang is Asset Tangibility, Reg is Regulations, and GDP is Gross Domestic Product.

To ensure the validity of the results, the study applied dynamic techniques to eliminate the fixed effects and controlling autocorrelation, heteroskedasticity, and cross-sectional dependence. The lagged values of the dependent variables are therefore used as instruments to control for this endogenous relationship. These instruments are often referred to as “internal instruments” because they are used from the existing econometric model [10]. Wintoki et al. [56] used two lags of the dependent variables and found that two lags are sufficient to capture the persistence of the dependent variable (e.g., firm performance). In addition, to avoid potential data loss due to the internal transformation of the first-stage GMM, Arellano and Bover [53] suggested using a second-order transformation (two-stage GMM).

The study estimated the panel dynamic model using system GMM by following the rules of thumb by Bond et al. [57]. Firstly, the study assessed the autoregressive model using pooled OLS and fixed effects approach. According to Bond et al. [57], the pooled OLS estimate ∅ in Eq (2) is considered an upper bound while its corresponding fixed effect estimate is considered a lower bound. We also estimated the difference GMM and compared its ∅ estimates with the lower bound estimate of the fixed effect regression. Bond et al. suggests that if the ∅ estimate of the difference GMM is lower than the lower bound, we ignore the difference GMM and rather use the system GMM. As can be observed from Table 2 below, not all the ∅ estimates of the difference GMM were higher than the lower bound values of the fixed effect estimates, and so the study used the system GMM in this study.

**Table 2:** Deciding between difference and system GMM. Comparing difference GMM estimation coefficient lag dependent variables to lower bound (fixed effect) estimates to decide whether to use difference GMM or system GMM.

OLS				Fixed Effect			One step difference GMM		
VARIABLES	ROA	ROE	OSS	ROA	ROE	OSS	ROA	ROE	OSS
L.ROA	0.482***			0.150***			0.365***		
	(0.031)			(0.040)			(0.103)		
L.ROE		0.463***			0.226***			0.480***	
		(0.031)			(0.040)			(0.045)	
L.OSS			0.045***			0.012*			-0.001
			(0.009)			(0.007)			(0.005)

**Source**: Authors’ regression results

**Notes**: In deciding between the difference and system GMM, the rule is to maintain (drop) the use of difference GMM if the lag coefficient estimates of the difference GMM are higher (lower) than the lag coefficient estimates of fixed effects. From the table above, not all the lag coefficients of the difference GMM estimates are higher than the lower bound (fixed effect) coefficient estimates. For example, the lag coefficient estimate of Operational Sufficiency (OSS) in the difference GMM is lower than its corresponding fixed effect estimate. As a result of that, system GMM is rather employed in this study instead of difference GMM.

We use the two-step system GMM model to prevent excessive data loss because it provides more efficient and consistent estimates for the coefficients concerned [58]. In addition, the two-step system GMM model subtracts the mean of all available future observations of a particular variable [10]. In all regressions, the lags of dependent variables were statistically significant to justify our use of the system GMM. GMM estimators for panel data analysis were generally robust to deviations in the data generation process underlying violations of homoscedasticity and normality to the extent that they were asymptotically normal. The GMM estimator allows for arbitrary heteroskedasticity and serial dependence in large N and small T panels using the optimal weighting matrix [55, 59]. This condition was shown in our case since the research had 735 observations and T = 7 years.

## Result and discussion

### Summary statistics

Table 3 shows summary statistics of the variables used in the analysis. The descriptive statistics are for both dependent and independent variables over the seven years from 2011–2017. It can be seen from Table 3 that Return of Assets (ROA) has an average value of -0.3 percent, Return on Equity (ROE) has a mean value of 1.2 percent, and Operational Self-Sufficiency (OSS) has an average value of 106.5 percent. The means of the dependent variables in Table 3 (apart from ROA) suggest that MFIs have positive average earnings indicating sufficient operating revenues to cover their various costs. The mean of ROA (-0.3 percent) is an indication that MFIs are not able to efficiently utilize their assets to generate returns. OSS is the most efficient variable to measure financial viability, and it is the ability to provide services to customers without depending on subsidies. It also provides more information than other financial performance variables such as ROA and ROE [60]. The positive value of ROE means that MFIs receive returns from investment to generate profit for each unit of shareholders’ funds. The negative value of ROA means that MFIs in the industry, on average are inefficient.

**Table 3:** Summary statistic.

Variables	Obs	Mean	Std. Dev.	Min	Max
**ROA**	735	-0.003	0.077	-0.9918	0.233
**ROE**	735	0.012	0.316	-5.035	1.881
**OSS**	735	1.065	1.179	0	31.964
**Depth**	735	0.959	1.392	0	19.494
**Breadth**	735	9.196	1.603	4.779	13.530
**PFB**	735	0.480	0.225	0	1
**Equity**	735	0.301	0.336	-1.498	7.116
**Deposits**	735	0.411	0.222	0	1.030
**Borrowings**	735	0.169	0.151	0	0.721
**Firm Size**	735	15.896	2.151	0	22.036
**PAR30**	735	0.069	0.172	0	3.030
**Asset Tangibility**	735	0.066	0.056	0	0.537
**GDP**	735	0.047	0.030	-0.206	0.207
**CPFS**	735	0.259	0.180	-0.161	1.852
**HDI**	735	0.491	0.062	0.325	0.699
**Age**	735	0.838	0.369	0	1
**Regulation**	735	0.933	0.250	0	1

Source: Authors’ compilation

The average depth of outreach is 95.9 percent indicating that MFIs accomplished a better service to the poor in Sub-Saharan African countries. This result suggests that MFIs in the SSA region have achieved a better depth of outreach and tend to serve lower-income clients.

The information presented in Table 3 indicates that most of the MFIs in Sub-Saharan Africa (SSA) are highly leveraged, as shown by the mean of 30.1 percent of equity. The results also show that MFIs use less internal resources and rather use more external resources to finance their activities in the industry. From the result (Table 3), it can be concluded that men represent the majority of borrowers (52 percent) from the MFIs in SSA compared to women (PFB of 48 percent). Although the difference is not too wide, this result shows that women do not contribute enough to the development of MFIs in SSA probably because of the socio-cultural context, which gives less power to the female or lack of entrepreneurs training to women. This result suggests that MFIs in SSA have not yet achieved one of the important goals of microfinance which is to promote women’s economic development. In addition, this result suggests that the microfinance sector extends credit to fewer women than men, thus making fewer women financially independent than men.

Table 4 shows the result of the Variance Inflation Factor (VIF), which measures the degree of multicollinearity among the explanatory variables. As depicted in Table 4 below, the overall mean VIF value is 1.40, suggesting no general multicollinearity problem among the explanatory variables in the study. The range of VIF for the variables is from 1.01 to 1.94.

**Table 4:** Variance Inflation Factor (VIF).

Variables	VIF	1/VIF
**Deposits**	1.9400	0.5155
**Firm Size**	1.8300	0.5471
**Breadth**	1.7900	0.5571
**Borrowings**	1.5400	0.6508
**PFB**	1.4300	0.6988
**Equity**	1.4100	0.7101
**Asset Tangibility**	1.1700	0.8518
**Depth**	1.1300	0.8865
**CPFS**	1.0800	0.9262
**Regulation**	1.0400	0.9582
**GDP**	1.0100	0.9864
**Mean VIF**	1.4000	

The study performed the Pearson correlation matrix to reaffirm the non-existence of multicollinearity among our variables (see, Table 5) below. According to Kennedy (2008), as cited in Ibrahim et al. [61], the correlation among variables should not exceed 0.8, and those two variables with a correlation coefficient of above 0.8 cannot be used together in the same regression. In Table 5, we present the result of the Pearson correlation Matrix, which shows that no correlation value surpassed 0.8. The study concludes that our data is devoid of multicollinearity problems.

**Table 5:** Pearson matrix.

	ROA	ROE	OSS	Depth	PFB	breadth	EQUITY	DEPOS	BORR	Size	Tang	GDP	CFPS	Reg
**ROA**	1													
**ROE**	0.800***	1												
**OSS**	0.158***	0.121***	1											
**Depth**	0.0300	0.0069	0.00524	1										
**PFB**	-0.0617	-0.0207	-0.0367	-0.156***	1									
**Breadth**	0.109**	0.0748*	0.0112	-0.0394	0.450***	1								
**EQUITY**	0.0311	-0.0124	0.0007	-0.0872*	0.206***	0.00241	1							
**DEPOS**	0.153***	0.161***	0.0364	0.276***	-0.252***	0.00742	-0.280***	1						
**BOR**	-0.0782*	-0.116**	-0.0164	-0.141***	0.197***	0.220***	-0.138***	-0.440***	1					
**Size**	0.199***	0.159***	0.0407	0.213***	0.0509	0.483***	-0.190***	0.412***	0.0507	1				
**Tang**	-0.140***	-0.105**	-0.0384	-0.0790*	0.0357	-0.000695	0.359***	-0.0714	-0.0661	-0.146***	1			
**GDP**	0.00855	0.0263	0.0557	0.0315	0.0586	0.0428	0.0424	-0.00316	0.0151	0.0264	0.0183	1		
**CFPS**	-0.000934	-0.0181	-0.0217	-0.0550	0.0671	0.177***	-0.108**	-0.0294	0.178***	0.151***	-0.0562	-0.0739*	1	
**Reg**	-0.0916*	-0.0210	-0.143***	0.0617	-0.0482	-0.0798*	-0.0389	0.165***	-0.151***	0.0356	0.0158	-0.00426	-0.0297	1

*** p<0.01

** p<0.05

* p<0.1

### Regression result

Table 6 displays the GMM results. Our regression result shows that the depth of outreach is positively and significantly related to profitability, Return on Asset (ROA) and Return on Equity (ROE) at the 1 percent and 5 percent significant levels respectively, hence a 1 percent increase in depth of outreach will lead to 0.2 percent increase in ROA and 1.7 percent increase in ROE for MFIs. This result indicates that MFIs can trade-off well by reaching out to more poor people through loans/credit facilities to open or expand their businesses, of which repayment of loans are made on time, leading to MFIs achieving their objective of financial performance. This has helped reduce poverty among the poor people in sub-Saharan African countries and helped these MFIs cover the risks associated with their operations. This agrees with Cull et al. [16], who show evidence that MFIs can maintain the depth of outreach and remain profitable.

**Table 6:** Trade-off between financial and social performance.

VARIABLES	ROA	ROE	OSS
**L.ROA**	0.450***		
	(0.004)		
**L.ROE**		0.480***	
		(0.025)	
**L.OSS**			0.021***
			(0.001)
**Depth**	0.002***	0.017**	-0.018***
	(0.000)	(0.007)	(0.001)
**PFB**	0.004	0.187***	-0.379***
	(0.005)	(0.068)	(0.028)
**Breadth**	0.002***	0.004	0.036***
	(0.001)	(0.009)	(0.004)
**Equity**	0.035***	0.216***	0.026*
	(0.002)	(0.071)	(0.016)
**Deposits**	0.090***	0.411***	-0.026
	(0.005)	(0.064)	(0.026)
**Borrowings**	0.083***	0.572***	-0.240***
	(0.003)	(0.066)	(0.029)
**Firm Size**	0.000	-0.014**	0.061***
	(0.000)	(0.006)	(0.003)
**Asset Tangibility**	-0.140***	-0.460	-0.072
	(0.009)	(0.289)	(0.089)
**GDP**	0.015	-0.622*	-0.993***
	(0.017)	(0.357)	(0.088)
**Regulation**	0.045***	0.117**	-0.049**
	(0.005)	(0.052)	(0.023)
**Constant**	-0.127***	-0.291***	0.030
	(0.007)	(0.094)	(0.042)
**Observations**	630	630	630
**Number of MFI**	105	105	105
**Year Dummies**	Yes	Yes	Yes
**Hansen p**	0.629	0.476	0.533
**AR(2)p**	0.325	0.265	0.107
**AR(1) p**	0.000104	0.0180	0.000864

Standard errors in parentheses

*** p<0.01

** p<0.05

* p<0.1

The result further shows a negative relationship between the depth of outreach and sustainability. An increase in the depth of outreach by 1 percent decreases Operational self-sufficiency (OSS) by 1.8 percent at the 1 percent significant level. This means that providing more credit facilities (average loans balance) to borrowers (clients) will lead to a decrease in MFIs financial sustainability in the form of increasing operational cost against its revenue. MFIs can serve growing numbers of poor people to reduce poverty levels but at the peril of their financial sustainability. This could further mean that MFIs need more support such as international aid, donations, and subsidies from investors and or government to sustain the industry. This concurs with Hermes et al. [31] that the depth of outreach in MFIs negatively affects financial sustainability, but Paxton [62] rebuffs the notion of any trade-off between financial sustainability and outreach.

Percentage of Female Borrowers (PFB) is also positive and significantly related to profitability (ROA and ROE). A unit change in PFB will lead to the profitability of MFIs in SSA countries by 18.7 percent. This means that MFIs can trade-off by giving more credit facilities to women to start or expand their businesses. This helps increase the profit of MFIs in SSA and allows the reduction of poverty among the poor women within SSA who are most vulnerable in society. This confirms the study of Cull et al. [16] that increasing profitability of MFIs is associated with a decreasing trend of outreach to the poor. Abdullah and Quayes [63] and Karanja [64] found a positive association between financial performance and outreach to women. However, Cull et al. [40] assert that the fraction of female borrowers might be lower in financially sustainable MFIs.

Breadth of outreach refers to the type or profile of clients served by the MFIs, and it represents the scale of MFI operations [45]. However, a high ratio of this variable indicates an increasing number of MFI operations and fewer poor clients being reached. This is consistent with institutionalists who believe that poverty reduction is related to the number of poor clients MFIs can serve [47].

Breadth of outreach is positively related to profitability and significantly associated with ROA and OSS. From Table 6 below, a unit change in breadth of outreach will increase the MFIs industry’s financial performance by 0.2 percent in term of ROA. This means that MFIs can reach the majority of borrowers, which has helped to increase the profit of MFIs. Thus, MFIs can operate and efficiently generate income from their assets to cover all the associated risks. The most important goal is to give customers more credit and be reassured that there is a high repayment rate.

In the case of breadth outreach and Operational self-sufficiency (OSS), a unit change in breadth outreach will lead to an increase OSS by 3.6 percent; this means that the more borrowers the MFIs can reach out to the higher of MFIs being able to cover its costs through balancing of its operations. MFIs are able to trade-off by providing microfinance services to clients in a profitable manner as well as ensuring the sustainability of the firms. This confirms Adhikary and Papachristou [25] assertion that the depth of outreach is positively related to financial performance, implying that financially sustainable expansion of microfinance can achieve social objectives at an acceptable level of credit risk.

Our result also shows that the percentage of female borrowers (PFB) and depth of outreach are negative and significantly correlated to sustainability at the 1 percent significant level. The result indicated that increasing the PFB through increasing the average credit balance per client leads to a decline in poverty levels among females in SSA, which negatively affects the sustainability of the MFIs industry. This means that an increase in the percentage of MFIs outstanding debt reduces operational revenue. Based on that, MFIs face financial problems in meeting the growing demand for credit with some resorting to going to the capital market to borrow at higher interest rates which increases the portfolio exposure of the MFIs. This challenge affects the sustainability of MFIs in SSA countries. This finding is in tandem with Zainuddin et al. [30] who found a negative correlation between PFB and depth of outreach and sustainability, which they attributed to cultural factors.

However, NGO [65] found a positive relationship between the depth of outreach, breadth of outreach, and sustainability. A unit change in breadth of outreach will lead to a 3.6 percent change in MFIs sustainability in SSA. As MFIs extend credit to more people and ensure prompt repayment of such facilities, they become sustainable.

Our results show a significant negative correlation between depth of outreach and Operational self-sufficiency (OSS) and between the percentage of female borrowers (PFB) and OSS. These results contradict findings in the existing literature, which show a positive relationship. Our result does not support the evidence from existing literature that reports a positive and significant relationship between social performance and financial performance when we consider financial performance as profitability. We did not find a significant positive correlation between social performance (depth of outreach and PFB) and financial performance (OSS) using the GMM estimation approach.

Moreover, the effect of the financial equity ratio is observed to be positive and significantly related to profitability and sustainability. This result indicates that MFIs perform better (profitable and sustainable) with more equity capital. Examining the other control variables, we find that financial deposits are positive and significantly related to profitability (ROA and ROE) at the 1 percent significant level. The result suggests that taking deposits makes MFIs profitable even though it insignificantly hurts their operational self-sufficiency. It encourages MFIs to promote a savings culture among their clients who use them as collateral for loans advanced to them.

Borrowings are positive and significantly related to profitability at the 1 percent significant level. A 1 percent change in borrowings will lead to the profitability of MFIs in SSA countries by 8.3 percent. It shows that borrowing by clients helps MFIs to achieve profitability. The result demonstrates that MFIs borrow at preferential rates, then lend to clients and make a profit that covers their expenses and becomes profitable. It may also suggest that MFIs can pass on any interest cost associated with borrowed funds to their clients. Borrowings are negative and significantly associated with sustainability at the 1 percent significant level. It shows that increasing MFIs borrowing does not help them to achieve sustainability. This could be explained by the fact when lending to clients, MFIs focus on the rich client’s segment of the population to the neglect of the poorest who are perceived not to have the ability to repay their loans.

Asset Tangibility is observed to be negative and significantly related to Return on Asset. This result shows that MFIs invest more in assets than in loans to clients resulting in a decline in profit. GDP is negative and significantly related to profitability as well as sustainability. A decrease in GDP tends to affect the purchasing of goods and services, thus affecting MFI clients, causing them to default on the repayment of their loans. These loan defaults affect the profitability of MFIs. Regulated MFIs are positive and significantly related to profitability, and negative significantly associated with sustainability. Strictly regulating MFIs make them more financially profitable but not sustainable. This may be due to their over-concentration not on the poorest in society.

### Robustness check

To check the robustness of the result, we used the second lags of the variables. This represents performance in the year before (the previous year) in Table 7. Using the second lag of the variables is to check whether the results of the two previous periods are similar to the current period results. The lags are included as explanatory variables in our GMM estimation. Table 7 shows that the depth of outreach is negatively and significantly associated with Operational self-sufficiency (OSS) at the 1percent significance level. Also, the Percentage of Female Borrowers (PFB) is negative and significantly related to OSS at the 5 percent significance level. These results suggest that social performance is negatively associated with financial performance, as shown in our result. In addition, the breadth of outreach is negatively and significantly associated with Return of Equity (ROE) at the 1percent level. In contrast to existing literature, we do not find a significant positive relationship between social and financial performance when considering OSS as financial performance and taking into account the past (lag of at least two years in time). This shows the weakness of the table of results indicating the reciprocity between MFIs dual missions over time [66, 67].

**Table 7:** Robustness between social and financial performance with 2 lag.

VARIABLES	ROA	ROE	OSS
**L.ROA**	0.559***		
	(0.015)		
**L2.ROA**	-0.041***		
	(0.015)		
**L.ROE**		0.520***	
		(0.003)	
**L2.ROE**		-0.186***	
		(0.004)	
**L.OSS**			0.426***
			(0.014)
**L2.OSS**			0.018***
			(0.001)
**Depth**	-0.002	-0.001	-0.007***
	(0.001)	(0.001)	(0.002)
**PFB**	-0.002	0.089***	-0.087**
	(0.007)	(0.016)	(0.036)
**Breadth**	0.010***	-0.033***	0.004
	(0.001)	(0.003)	(0.004)
**Equity**	0.041***	-0.039***	-0.053***
	(0.007)	(0.011)	(0.016)
**Deposits**	0.067***	0.028	-0.044
	(0.009)	(0.018)	(0.027)
**Borrowings**	0.137***	-0.103***	-0.084***
	(0.013)	(0.017)	(0.029)
**Firm Size**	-0.000	0.026***	0.048***
	(0.001)	(0.002)	(0.003)
**Asset Tangibility**	-0.101***	-0.546***	-0.320***
	(0.029)	(0.038)	(0.083)
**GDP**	0.152***	0.257***	-0.509***
	(0.041)	(0.047)	(0.117)
**Regulation**	0.030***	0.203***	0.013
	(0.005)	(0.017)	(0.026)
**Constant**	-0.180***	0.000	-0.109***
	(0.016)	(0.000)	(0.039)
**Observations**	525	525	525
**Number of MFI**	105	105	105
**Year Dummies**	Yes	Yes	Yes
**Hansen p**	0.278	0.497	0.272
**AR(2)p**	0.713	0.189	0.693
**AR(1)p**	0.00217	0.137	0.00210

Standard errors in parentheses

*** p<0.01

** p<0.05

*p<0.1

Furthermore, the equity ratio has a significant negative correlation with OSS, suggesting that an increase in equity hamper the sustainability effort of MFIs. Also, deposits are significant and positively related to ROA. Intuitively, MFIs need more deposits when they want to become profitable. Borrowings show a negative and significant relationship with OSS. This suggests that an increase in borrowings is associated with decreased sustainability. The results also show a significant positive correlation between GDP and profitability (ROA and ROE) and a negative and significant relation between GDP and sustainability (OSS). This indicates that an increased GDP positively impacts MFIs profitability and a negative effect on sustainability.

To further check robustness, the study classified MFIs into different types (NBFI, NGOs, Credit Union, and Banks) and applied a seemingly unrelated regression (SUR) to evaluate the reciprocal correlation between their dual missions. Table 8 reports our analysis for four subsamples. The NBFI subsample shows a negative and significant reciprocal relation between social (depth of outreach, PFB) and financial performance (OSS) at the 1 percent significance level. This demonstrates that better social performance is not associated with better financial performance. Rather, it indicates that social performance (breadth of outreach) is positive and significantly related to OSS. The result further shows that for NBFI to improve the outreach of their operations, they should focus on the breadth of outreach.

**Table 8:** Seemingly unrelated regressions (SUR) regression result for different types of MFIs.

	NBFI			NGOs			Credit Union			Banks		
VARIABLES	ROA	ROE	OSS	ROA	ROE	OSS	ROA	ROE	OSS	ROA	ROE	OSS
**Depth**	-0.000	-0.011	-0.047***	0.015	-0.029	-0.023	0.006	-0.001	0.086***	-0.004***	-0.012**	-0.035
	(0.007)	(0.035)	(0.018)	(0.015)	(0.052)	(0.053)	(0.004)	(0.017)	(0.027)	(0.001)	(0.005)	(0.081)
**Breadth**	0.005	0.001	0.052***	0.007	-0.000	-0.000	0.000	0.009	-0.004	0.003	0.012	0.178
	(0.006)	(0.029)	(0.015)	(0.005)	(0.016)	(0.017)	(0.002)	(0.010)	(0.015)	(0.002)	(0.010)	(0.162)
**PFB**	-0.064*	0.020	-0.310***	-0.042**	-0.137*	-0.014	-0.041***	-0.130*	-0.107	-0.002	-0.144	-0.893
	(0.037)	(0.172)	(0.088)	(0.021)	(0.071)	(0.072)	(0.015)	(0.070)	(0.108)	(0.026)	(0.109)	(1.789)
**Equity**	0.057**	0.042	0.211***	0.035***	0.068*	0.100**	-0.009	-0.060	0.103	0.255***	0.490**	-6.350**
	(0.026)	(0.119)	(0.060)	(0.012)	(0.040)	(0.040)	(0.027)	(0.125)	(0.192)	(0.045)	(0.190)	(3.113)
**Deposits**	0.052	0.163	0.353***	0.037	0.183*	0.066	0.021	0.015	0.154	0.046	0.167	-4.697*
	(0.042)	(0.196)	(0.100)	(0.028)	(0.093)	(0.095)	(0.023)	(0.106)	(0.163)	(0.041)	(0.173)	(2.825)
**Borrowings**	-0.112**	-0.696***	0.172	0.001	0.185	-0.072	0.049	-0.073	0.327	-0.037	-0.063	-5.496
	(0.050)	(0.229)	(0.117)	(0.034)	(0.115)	(0.118)	(0.031)	(0.143)	(0.219)	(0.052)	(0.217)	(3.557)
**Firm Size**	0.014***	0.048**	0.031**	0.007*	0.023*	0.056***	0.004**	0.018**	0.035***	0.000	0.003	0.020
	(0.005)	(0.024)	(0.012)	(0.004)	(0.013)	(0.013)	(0.002)	(0.009)	(0.013)	(0.002)	(0.007)	(0.120)
**Asset Tangibility**	-0.351***	-0.674	-0.633**	-0.287***	-0.697**	-0.671**	-0.049	-0.485*	-0.238	-0.168***	-0.790***	-1.709
	(0.127)	(0.585)	(0.298)	(0.088)	(0.297)	(0.304)	(0.054)	(0.247)	(0.380)	(0.059)	(0.247)	(4.048)
**GDP**	0.287	1.606*	1.064**	0.355*	0.006	1.790**	-0.195**	-0.651	-1.822***	-0.037	0.157	13.706
	(0.178)	(0.824)	(0.420)	(0.212)	(0.716)	(0.732)	(0.095)	(0.438)	(0.672)	(0.147)	(0.621)	(10.168)
**Regulation**	-0.013	-0.019	0.087	-0.028*	-0.015	-0.030	-	-	-	0.008	0.038	-4.977***
	(0.026)	(0.122)	(0.062)	(0.015)	(0.050)	(0.051)				(0.015)	(0.063)	(1.031)
**Constant**	-0.276***	-0.750**	-0.191	-0.158***	-0.195	0.187	-0.056**	-0.163	0.407**	-0.094**	-0.226	10.054***
	(0.066)	(0.303)	(0.154)	(0.049)	(0.165)	(0.168)	(0.025)	(0.117)	(0.179)	(0.038)	(0.159)	(2.597)
**Observations**	245	245	245	181	181	181	175	175	175	133	133	133
**R-squared**	0.181	0.125	0.295	0.216	0.130	0.224	0.264	0.195	0.304	0.459	0.229	0.231
**Year Dummies**	Yes	Yes	Yes	Yes	Yes	Yes	Yes	Yes	Yes	Yes	Yes	Yes

Standard errors in parentheses

*** p<0.01

** p<0.05

* p<0.1

In addition, the result for NGOs subsample shows a negative and significant association between social performance (PFB) and financial performance (ROA and ROE). However, our result indicates that outreach is insignificantly related to financial performance. It also demonstrates that for NGOs, there is a trade-off. Also, the result for credit unions confirms a positive and significant correlation between social performance (depth of outreach) and financial performance (OSS). It indicates that better social performance will always lead to better financial performance.

Moreover, for credit unions, the study found that the coefficient on financial performance (ROA and ROE) is negative and significantly related to social performance (PFB). This result indicates that increasing financial performance will lead to decreasing the number of female borrowers. The result for the bank further shows a negative and significant relationship between social performance (depth of outreach) and financial performance (ROA and ROE). This result demonstrates that better financial performance is related to worse social performance.

In summary, we find that the reciprocal correlation between social and financial performance depends on the type of MFIs and which variables we consider as social and financial performance. For example, for NBFI, Banks, and NGOs show a negative and significant correlation between social performance and financial performance. Credit unions seem to be more operational than NBFI, NGO, and Banks in transforming better social performance (depth of outreach) to better financial performance. The significant and negative relationship between social and financial performance demonstrates the existence of trade-off. Therefore the achievement of one of two objectives obliges the abandonment of the other.

Furthermore, managing MFIs with a dual mission is more complicated. NBFI, NGOs, and Banks are worse than credit unions at transforming better social performance (depth of outreach) into better financial performance outcomes. This can accentuate the mission drift of NBFI, NGOs, and Banks because their investors will pressure them for profit or market returns. Hence, NBFI, NGOs, and Banks can learn from the credit unions to become more operational in transforming social effect into financial profitability and sustainability.

The loans granted by NBFI, NGOs, and Bank officers are less performing than those of the credit unions. This suggests that loan officers have to receive more support through training to improve their performance and become profitable to reduce poverty and improve their social performance. The more efficient MFIs are, the better they will be able to cope with these trade-offs. However, the results shown by the different types of MFIs could help investors to have more information about the type of MFI over another and help the microfinance sector to be sustainable and efficient.

However, we observe that the breadth of outreach as social performance is positively and significantly associated with financial performance (OSS) when NBFI is a type of MFI. This result provides strong evidence that there is an optimal relationship between outreach and sustainability. In other words, it provides evidence of a trade-off between outreach and sustainability beyond the type of MFI. Managers should increase or create new services for the poor and increase their clientele to achieve a higher level of sustainability. The results demonstrate that both objectives can be achieved simultaneously.

Our findings suggest that the type of MFI can significantly impact the reciprocal correlation between MFIs social and financial performance. The decision made by managers or MFIs owners can affect how the dual mission can be accomplished. Our result sheds light on the types of MFI that meet expectations before investing. Additionally, the equity ratio is positive and significantly related to financial performance for NBFI, NGO. This suggests that the owners of those institutions in SSA should increase their funding. Deposits are negative and significantly associated with OSS for the Banks as MFI. This shows an increase in deposits impedes sustainability efforts pursued by Banks. Therefore, the Banks use their deposits for purposes other than lending to the poor, and also the interest rates that the Banks offer are adjudged to be very high by the clients. The borrowings ratio is negatively and significantly associated with profitability for NBFI. This result can be explained by the high cost of borrowing in SSA; as such NBFIs need to focus more on other types of financing than borrowings.

## Conclusion

The study examined the relationship between social performance and financial performance of MFIs in SSA from 2011 to 2017. The study used the MIX market database covering 105 MFIs in 26 countries in Sub-Saharan African countries. Social performance is analyzed using system GMM with Return on Equity (ROE), Return on Asset (ROA), and Operational Self-Sufficiency (OSS) as dependent variables. We explored three dimensions of outreach (breadth of outreach, depth of outreach, and percentage of women borrowers). A significant negative relationship between financial and social performance indicates that MFIs are unable to trade-off well. The increase in financial performance negatively affects social performance, which in turn affects MFIs sustainability. In contrast, a significant positive relationship suggests that both objectives can be accomplished together.

We conclude that different types of MFIs display differences in the trade-off between social and financial performances. Credit unions tend to show more efficiency in transforming social performance (depth of outreach) into consequent financial performance. Also, NBFI tends to be more efficient in transforming social performance (breath of outreach) into resulting financial performance. These results provide information on the sustainability of the different types of MFIs. NBFI, Banks, and NGOs show a negative and significant correlation between social performance and financial performance based on the depth of outreach and profitability (ROA and ROE) for Banks; depth of outreach, PFB and sustainability (OSS) for NBFI; and percentage of female borrowers (PFB) and profitability (ROA and ROE) for NGOs. These results confirm the existence of a trade-off. However, credit unions seem to be more operational than NBFI, NGOs, and Banks to transform better social performance (depth of outreach) to succeeding better financial performance. The trade-off between social performance and financial performance in the study is dependent on the variables selected for the analysis. This demonstrates how sensitive it is to the measurement of the effectiveness of mission drift. In addition, our results indicate that women should be a priority target in pursuing sustainability and poverty reduction.

We also conclude that deposit from clients, strict regulations, and increased client borrowings lead to MFI profitability but decreases sustainability in terms of depth and breadth of outreach. We observe that MFIs can become more sustainable if each type of MFI can learn from the other to ameliorate its weaknesses and that the type of MFI is a very important element for different decision-makers to better understand the importance of the relationship between social performance and financial performance to determine the existence of a compromise between the two objectives.

We further conclude that the capital structure of MFIs determines their profitability and sustainability. From our results, an increase in the debt component of MFIs’ capital structure produces significant increases in ROA and ROE compared to an increase in the equity component of the capital structure. However, an increase in the debt component of MFIs’ capital structure leads to unsustainability while higher equity component leads to sustainability. Hence, to sustain MFIs in SSA, their capital structure should be more equity than debt.

Finally, the study suggests the following recommendations: (1) MFIs should purposefully increase credit facilities extended to female borrowers since that will make them sustainable. (2) Management of MFIs should train staff periodically on effective strategies of identifying credit-worthy customers to sustain their business. (3) Management of MFIs should churn out innovative products and services that can lead to increased deposit mobilizations and many borrowers in their bid to increase profitability, and (4) Governments in Sub-Saharan African countries should provide increased financial support in the form of subsidies and tax holidays to MFIs operating in very deprived areas. This will enable them to achieve their social performance, thereby aiding them to offer credit to the poor in deprived rural areas at a reasonable cost.

The use of breadth of outreach, depth of outreach and percentage of female borrowers as a measure of social performance is a limitation in this study since the composition of social performance varies. For example, social performance can be measured by number of active borrowers, market share of borrowers, market share of number of borrowers adjusted by market share of assets, percentage of gross rural loan portfolio, etc. Future research should consider integrating household data with MFI-level data to investigate the actual impact of MFIs using direct measurements of customer’s poverty and wellbeing. Future research should also explore the use of other variables in assessing the social and financial performances of MFIs.
